# Safety of Combination TARE and SBRT in Hepatocellular Carcinoma: A Review of Literature & Single-Center Case Series

**DOI:** 10.3390/curroncol32090487

**Published:** 2025-08-31

**Authors:** Bahareh Gholami, Ali Afrasiabi, Andrew M. Moon, Ted K. Yanagihara, Hui Wang, Sandra Gad, Alex Villalobos, David M. Mauro, Hyeon Yu, Johannes L. du Pisanie, Nima Kokabi

**Affiliations:** 1 Division of Vascular and Interventional Radiology, Department of Radiology, University of North Carolina at Chapel Hill, Chapel Hill, NC 27599, USA; bahareh_gholami@med.unc.edu (B.G.); ali_afrasiabi@med.unc.edu (A.A.); hui_wang@med.unc.edu (H.W.); sandra_gad@med.unc.edu (S.G.); david_mauro@med.unc.edu (D.M.M.); hyeon_yu@med.unc.edu (H.Y.); lourens_dupisanie@med.unc.edu (J.L.d.P.); 2 Division of Gastroenterology and Hepatology, Lineberger Comprehensive Cancer Center, University of North Carolina at Chapel Hill, Chapel Hill, NC 27599, USA; andrew.moon@unchealth.unc.edu; 3 Department of Radiation Oncology, Lineberger Comprehensive Cancer Center, University of North Carolina at Chapel Hill, Chapel Hill, NC 27599, USA; tky@email.unc.edu

**Keywords:** hepatocellular carcinoma, transarterial radioembolization, stereotactic body radiation therapy

## Abstract

Hepatocellular carcinoma (HCC) is the most common primary liver cancer. At the time of diagnosis, many HCC patients are not candidates for surgical resection and are considered for other locoregional therapies, including transarterial radioembolization (TARE) and stereotactic body radiation therapy (SBRT). To date only a few studies have explored the safety and efficacy of combining TARE and SBRT. Therefore, we aimed to evaluate it. Patients who received both SBRT and TARE from 2016 to 2024 were retrospectively evaluated for treatment-related toxicity based on criteria for adverse events (CTCAE v4.0). Treatment response was evaluated by modified response evaluation criteria for solid tumors (m-RECIST). We identified 12 patients with median age of 66.5 (range: 40, 87) and median follow up of 12 months. The median time between TARE and SBRT was 6.5 months (range: 1.5 to 24). Following the second treatment, ALBI grade remined the same among all patients at 3-month post treatment compared to baseline. Baseline CP was A among all patients and remained unchanged during follow-up and no higher than grade 3 clinical or biochemical toxicity was seen. The objective response rate (ORR) among patients receiving treatment to the same lesion was 100%. The combination treatment was consistent with prior studies in which the combination of TARE and SBRT has been shown to have good local control with few cases of grade 3 toxicity. Our study demonstrates that treatment with TARE and SBRT was safe and effective among our small sample of patients.

## Simple Summary

Liver cancer is the sixth most commonly diagnosed cancer worldwide and the third leading cause of cancer-related mortality, and many patients with this cancer cannot have surgery. Treatments such as transarterial radioembolization (TARE) and stereotactic body radiation therapy (SBRT) are two common options but using both together is rare due to safety concerns. This study reviewed 12 patients who had both treatments. Their liver function remained stable, and there were no severe adverse events. When both treatments targeted the same lesion, the objective response rate (ORR) was 100%. These findings suggest that combining TARE and SBRT might be safe and effective, however larger sample and prospective studies need to be undertaken to confirm these findings.

## 1. Introduction

Liver cancer is the sixth most commonly diagnosed cancer worldwide and the third leading cause of cancer-related mortality, with more than 830,000 deaths reported in 2020 [1]. Hepatocellular carcinoma (HCC), which constitutes over 80% of liver cancer cases, is a major global health concern and ranks as one of the three leading causes of cancer-related deaths in 46 countries [2].

Based on the 2022 revision of the Barcelona clinic liver cancer (BCLC) guidelines, surgical resection, liver transplantation (LT), and ablative techniques, such as radiofrequency ablation (RFA), are primary treatment choices for early-stage HCC [3]. However, only a few of these patients are eligible for such curative interventions, mainly because of technical limitations, more advanced disease, poor liver function, or comorbidities, necessitating alternative locoregional therapies such as ablation, transarterial chemoembolization (TACE), transarterial radioembolization (TARE) with yttrium-90 microspheres, and stereotactic body radiation therapy (SBRT) [4]. Ablation can be used as an alternative to resection for patients with lesions of less than 3 cm as a bridge to transplant [3].

TARE and SBRT are two increasingly used radiation-based treatment methods for HCC. SBRT is a non-invasive, locally ablative treatment option for patients with HCC with high rates of local control and a favorable safety profile [5,6,7,8]. Local control (LC) following SBRT is high, with 5-year LC of ~80% in pooled analyses and ~90% for lesions <3 cm [9]. The RASER trial, a prospective study on solitary nodules ≤3 cm unsuitable for ablation, showed TARE’s efficacy with a 100% objective response rate, including 83% complete response and a durable median duration of response of 635 days [10].

TARE is an effective locoregional treatment that involves the administration of microspheres by catheterization of the hepatic artery. One-year LC after TARE is shown to be similar to SBRT (89% vs. 87%, *p* = 0.76) [11]. Despite the high efficacy of both treatment modalities, there are certain clinical scenarios in which treatment with TARE or SBRT alone may be incomplete or inadequate (e.g., large tumors and those with extensive abutment to adjacent sensitive organs). In these cases, a combination of TARE and SBRT may offer a bimodal treatment option to maximize LC. However, a 2021 consensus statement advised against this combination treatment due to the concerns about radiation toxicity [12] which resulted in limited outcome data for the use of combined TARE and SBRT in patients with liver cancer [13,14,15]. Most available studies have small sample sizes, with one including only patients with portal vein tumor thrombosis [15]. Furthermore, the absence of a commercial method for composite dosimetry of TARE and SBRT raises concerns about potential radiation overdosing and hepatotoxicity in patients receiving this combined treatment [13,16]. Radiation induced liver disease (RILD) can present as abnormal liver enzyme levels, change in CP score, biliary strictures, or other gastrointestinal complications [17,18].

In light of this background, the aim of our study is to review the current literature on the combination TARE and SBRT for HCC treatment and outline our single institution case series of patients treated with combination of SBRT and TARE across time to assess their toxicity and radiological response to treatment.

## 2. Materials and Methods

The medical records of patients with HCC from 2016 to 2024 at UNC hospitals were retrospectively reviewed and 12 patients over 18 years old treated with SBRT after TARE or TARE after SBRT were identified.

The inclusion criteria were patients having HCC who had clinical follow-up after the last procedure for at least 3 months. Exclusion criteria were having metastatic cancer to the liver and not having at least three-month post treatment follow up data. All patient data were handled in compliance with the health insurance portability and accountability act (HIPAA). Protected health information (PHI) was de-identified prior to analysis, and access to data was restricted to authorized study personnel. This study received approval from our institutional review board.

For SBRT, all patients received a full evaluation that included lab work and imaging scans. The detailed treatment parameters have been published previously [19]. Briefly, all patients underwent a 4-dimensional computed tomography simulation scan in a supine position with customized immobilization. Target definition was based on all available imaging, including multi-phase contrast enhanced MRI fused with planning CT images. Treatment was typically delivered by CyberKnife^®^ Robotic Radiosurgery System (Accuray Incorporated, Sunnyvale, CA, USA) with fiducial marker placement, but could be delivered via Linear Accelerator (LINAC, Accuracy Incorporated, Sunny-vale, CA, USA) using a free-breathing or expiratory-breath hold technique, with or without fiducial marker placement. Total radiation dose for each patient was 45 Gy over 3 or 5 fractions, or 25 Gy over 5 fractions with a requirement that at least 700 mL of healthy liver receive less than 15 Gy. To calculate the functional liver volume, liver regions that had previously had TARE treatment were marked out and subtracted from the total liver volume.

Before the TARE procedure, all patients received a full evaluation such as laboratory tests, cross-sectional imaging studies, and a pre-TARE mapping angiography with technetium-99 macroaggregated albumin, as described previously [20,21,22].

Likewise for TARE, all patients received a full evaluation that included laboratory work such as liver function tests, complete blood count, creatinine, imaging scans, and a pre-TARE mapping angiography with technetium-99 macroaggregated albumin, as described previously [19,20,21]. Glass-based (Theraspheres, Boston Scientific, Marlborough, MA, USA) or resin-based (Sirtex Medical, Woburn, MA, USA) yttrium-90 (Y90) radioembolization were performed at the discretion of the treating interventional radiologist. Post Y90 single photon emission computed tomography (SPECT)/CT was performed to ensure appropriate targeting of tumors. Post Y90 dosimetry was also performed using MIM Sureplan^®^ (MIM Software, Cleveland, OH, USA). After the procedure, all patients were managed with a protocol that included antiemetics, analgesics, IV hydration, and preventive proton-pump inhibitors, aligning with methods previously reported [20].

The Child-Pugh (CP) score, ALBI score, and grade 3+ adverse events as defined by the common terminology criteria for adverse events (CTCAE v5.0) were used to measure toxicity. These adverse events included both biochemical lab abnormalities such as abnormal levels of alkaline phosphatase (ALP), alanine aminotransferase (ALT), aspartate aminotransferase (AST), albumin, or clinical events such as gastrointestinal adverse effects, bile duct stenosis, and liver failure, all determined by chart reviews during three-month interval visits after both TARE and SBRT.

Continuous variables were summarized by the median and range (minimum and maximum). The main outcomes evaluated were toxicity, assessed by CP and ALBI scores, as well as radiological treatment response based on modified RECIST (mRECIST) [23]. Python version 3.11.8 was used for plotting the data. According to treatment response, objective response rate (ORR) was defined as the percentage of patients receiving complete response (CR) or partial response (PR).

## 3. Results

### 3.1. Patient Characteristics

We identified 12 patients with median follow up of 12 months over the age of 18 years with HCC who were treated with SBRT after segmental TARE or vice versa from 2016 to 2024. Median age of the patients was 66.5 years (range: 40, 87) and 75% of them were male. Baseline characteristics of all the patients are shown in Table 1. All the patients had a CP score of A. Most of them had an Eastern cooperative oncology group (ECOG) performance status of 0 (58.3%). The majority were Barcelona clinic liver cancer (BCLC) grade C (41.6%) or A (33.3%). A total of 50% of patients had multifocal HCC. In terms of ALBI grade, 41.7% had grade 1 and 58.3% had grade 2. Most patients did not have prior locoregional treatments for HCC (41.7%). Almost all of them had glass-based Y90 treatment (91.6%). Post treatment dosimetry for TARE showed a median tumor dose of 520.25 Gy (range: 220, 1892) and median segment dose of 215.6 Gy (range: 93, 538). For SBRT, most patients (*n* = 10) received a total dose of 45 Gy, delivered at 15 Gy per fraction over 3 fractions using CyberKnife. Two patients received LINAC-based SBRT: one with a total dose of 25 Gy at 5 Gy per fraction over 5 fractions, and another with a total dose of 45 Gy at 9 Gy per fraction over 5 fractions. Nine patients received SBRT after TARE and three received TARE after SBRT. The median time between SBRT and TARE was 6.5 months (range: 1.5 to 24 months). Three patients received treatments to the same lesion previously treated with TARE or SBRT, and nine received treatments to a different lesion. Among our patients four had previous locoregional treatments. Patient 1 (multifocal HCC): initial TARE to segments 5 and 8 (partial response). Nine months later, repeat TARE to a new lesion in segment 4, followed by SBRT to the recurrence in segment 4. Patient 2: initial TARE to segment 8 (stable disease), followed by repeat TARE to the same lesion (partial response). Subsequent SBRT to the same lesion resulted in complete response. Patient 3: two rounds of TACE to segment 4A (first: stable disease; second: partial response), followed by TARE to the same lesion (partial response). Later, SBRT to a new lesion in segment 7 (partial response). Patient 4 (lesion in segment 4B): initial ablation (complete response), followed by recurrence treated with TARE. Finally, SBRT to a different lesion.

### 3.2. Toxicity Following Combination Treatment

**Figure 1:**
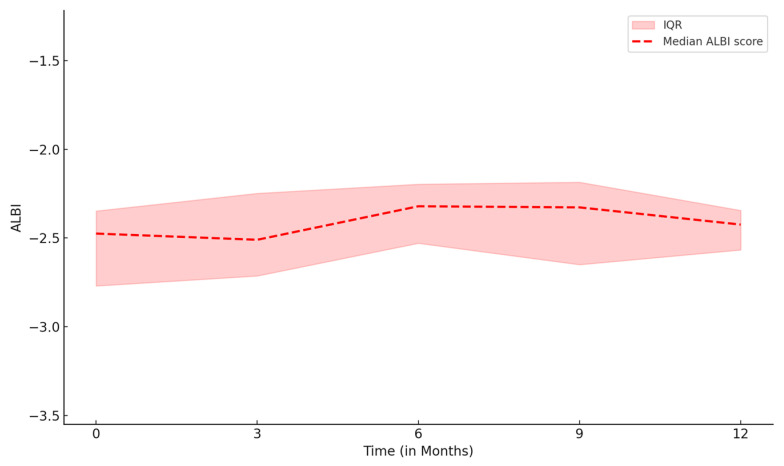
ALBI scores (median, interquartile range (IQR)) plot after the last treatment.

Following the last treatment with either TARE or SBRT, ALBI grade remined unchanged among all patients at 3-month post treatment compared to baseline ALBI grade. At six-month post treatment, three patients experienced decrease in ALBI grade from grade 2 to 1, while the rest of them remained the same ALBI grade as three-month post treatment. The median ALBI scores over time are shown in Figure 1.

All of the patients had a CP score of 5 at baseline which remained unchanged during follow up. There was no higher than grade 3 clinical or biochemical toxicity during the 12-month follow-up. Three patients experienced grade 2 toxicity. One patient had an event of grade 2 increase in creatinine level, one had an event of grade 2 increase in ALP, and one had an event of grade 2 decrease in albumin levels. Six patients had an event of grade 1 LFT increase. One patient had an event of grade one increase in bilirubin, and another had an event of grade 1 increase in INR (Table 2).

## 4. Discussion

Combination therapy with locoregional treatments has been under-investigated. There is evidence that combination of TACE with SBRT is safe and effective and patients with this combination had higher one- and three-year overall survival (OS) compared to TACE alone [24]. Patients undergoing this combination treatment in randomized control trials (RCTs) have also shown to have improved progression-free survival (PFS), objective response rate (ORR), and OS compared to those receiving sorafenib treatment [25].

Combination treatment with TARE and SBRT is being more considered by radiation oncologists however only a handful of studies have evaluated the safety and efficacy of this approach. For instance, Liu et al. conducted a retrospective study including 12 patients with liver cancer who had portal vein tumor thrombosis (PVTT). These patients received a median Y90 dose of 104.3 Gy and median SBRT dose of 32.5 Gy and had no late toxicities; however, seven patients experienced acute grade 1 toxicities. Based on RECIST criteria in their study, the CR rate was 8%, the PR rate was 42%, the SD rate was 33%, and the PD rate was 17%. Additionally, local control was 83% and 1-year OS was 55% with a median OS of 14 months [15]. In another study by Hardy-Abeloos et al. including 68 patients with HCC who received SBRT after TACE and 31 patients who had SBRT after TARE, the incidence of CTCAE grade ≥3 toxicities, local control of tumor, and OS were similar between the SBRT/TARE and SBRT/TACE groups; however, the best ORR was 66% in the SBRT/TARE group which was significantly lower compared to 89% in the SBRT/TACE group. Among patients receiving SBRT after TARE, the incidence of any grade ≥3 CTCAE toxicity was 9.4% (3 out of 31). Three patients in this group experienced acute hepatic failure [14]. Another study explored the dosimetric factors associated with hepatotoxicity. In this study the average liver doses from external beam radiation therapy (EBRT) and radioembolization (RE) were reported as 4.40 Gy and 57.9 Gy, respectively. Patients with hepatotoxicity (≥grade 2) showed significantly elevated EBRT mean liver doses (7.96 ± 8.55 Gy) compared to those without (1.62 ± 3.39 Gy; *p* = 0.037). In the multivariate analysis, the percentage of liver volume receiving ≥30 Gy (V30) was the strongest predictor of hepatotoxicity and those who surpassed V30 of 13% experienced hepatotoxicity. Additionally, two fatal cases of radiation-induced liver disease were linked to the highest EBRT mean liver doses (20.9 Gy and 23.1 Gy) [13].

Our study’s small sample size makes it exploratory in nature, and the results should be interpreted with caution. Our results align with prior studies suggesting that combined TARE and SBRT can be delivered with a favorable safety profile [15]. Our results are consistent with Liu et al. [15], who reported no late toxicities in their cohort of 12 patients with portal vein tumor thrombosis. Likewise, we did not observe any grade 3 or higher adverse events over a median follow-up of 12 months. Also, CP scores and ALBI grades at 3 months post-treatment remained unchanged which points to the safety of this combination. The only toxicities that occurred in our study were CTCAE grade 1 or 2. Contrary to our results, Hard-Abeloos et al. reported that 3 out of 31 patients treated with SBRT post-TARE experienced CTCAE grade 3 adverse events which we did not see in our patients [14].

Regarding the efficacy, we observed 100% objective response rate (ORR) among three patients who received TARE and SBRT to the same lesion. This suggests that targeting the same lesion with both modalities might enhance tumor response to treatment, due to a complementary effect of the two radiation-based therapies. However, this sample is too small to make conclusions and further large sample studies are required to confirm the efficacy. A key feature in this study is its inclusion of patients treated with TARE and SBRT to different lesions. These patients represent real-world challenge of multifocal HCC management, and the preserved liver function highlights the application of this combination treatment in clinical practice.

Our study’s primary limitations include its small sample size and potential risk of type II error. Additionally, this study did not include a control group. Furthermore, patients were carefully selected and therefore may represent a population at lower risk for post-treatment adverse events. Lastly, with any retrospective study there is a possibility of outcome misclassification.

## 5. Conclusions

Evidence on combination of TARE and SBRT is limited but generally favorable: retrospective cohorts show high local control and acceptable safety, with few cases of grade 3 hepatotoxicity after this combination. Consistently, in our study, this combination of SBRT and segmental TARE, either to the same area of the liver or different areas of the liver, appears to be safe. Larger, ideally prospective studies are needed to confirm this result.

## Figures and Tables

**Table 1:** Summary of patient’s baseline characteristics.

	Count (%)/Median (Range)
Age, year *	66.5 (40, 87)
Male sex	9 (75%)
Cirrhosis etiology	
HCV/alcohol	1 (8.3%)
MASLD ^¥^/alcohol	2 (16.6%)
MASLD/HCV	1 (8.3%)
MASLD	2 (16.6%)
HCV	3 (25%)
HCV/HBV	1 (8.3%)
No cirrhosis	2 (16.6%)
Prior liver directed therapies:	
None	8 (66.7%%)
RFA **	1 (8.3%)
TARE	2 (16.7%)
TACE/TACE	1 (8.3%)
First treatment in sequential treatment	
TARE	9 (75%)
SBRT	3 (25%)
ECOG:	
0	7 (58.3%)
1	4 (33.3%)
2	1 (8.3%)
Baseline BCLC class	
A	4 (33.3%)
B	3 (25%)
C	5 (41.6%)
Baseline CP	
A	12 (100%)
Baseline ALBI grade	
1	5 (41.7%)
2	7 (58.3%)
Baseline tumor burden:	
Single lesion <2 cm	0
Single lesion >2 cm	2 (16.6%)
2–3 lesions all <3 cm	4 (33.3%)
Multifocal	6 (50%)
Portal vein invasion	0
Metastatic spread	0
Time between TARE and SBRT (months)	6.5 (1.5 to 24)
Treatment to the same lesion	
Yes	3 (25%)
No	9 (75%)
Y90 delivery method	
Glass	11 (91.6%)
Resin	1 (0.4%)
Y90 segment dose (Gy)	215.6 (93,538)
Y90 tumor dose (Gy)	520.25 (220, 1892)
SBRT dose (Gy)	45 (25, 45)

* Continuous variables are presented as median (min, max) and categorical variables as count (percentage), ** Radiofrequency ablation, ^¥^ Metabolic-associated steatotic liver disease.

**Table 2:** Individual Patient Characteristics.

Gender	Age	Cirrhosis Etiology	Response	First Seg Treated	Second Seg Treated	Same Lesion Targeted	Baseline CTP Class	Baseline BCLC	Baseline ECOG	Baseline ALBI Grade	Y90 Segment Dose	Y90 Umor Dose	SBRT Dose Total (Gy)	Toxicities After Last Treatment
Male	67	NAFLD/HCV	SBRT: SD TARE:PR	7	7	Yes	A	A	0	1	143	377	45	Grade 2 Cr ^1^ ↑ Grade 1 ALP ^2^ ↑
Female	54	NAFLD	SBRT:SD TARE:CR	6	4	No	A	B	0	2	160	385	45	Grade 1 AST ^3^ ↑ Grade 2 ALP ↑ Late Grade 1 Bili ^4^ ↑ and Alb ^5^ ↓
Male	69	HCV	SBRT: PR TARE: CR	7	4	No	A	B	0	1	220	387	45	Grade 1 AST ↑, Grade 1 ALT ^6^ ↑
Male	66	HCV	TARE:PR SBRT: PR	5	2/3	No	A	B	1	1	538	1204	45	None
Male	55	HCV/alcohol	TARE:PR SBRT: SD	4	7	No	A	A	1	2	102	226	45	None
Female	50	HCV/Alcohol	TARE: PD SBRT: SD	5	Portal nodes	No	A	A	0	1	292.7	959.5	25	Grade 2 Alb ↓ grade 1 AST ↑
Male	66	HCV/HBV	TARE: PR SBRT: PR	6/7	5	No	A	C	1	1	177.9	342.3	45	none
Male	68	HCV	TARE: SD SBRT: CR	4a	4a	Yes	A	B	0	1	93	220	45	Grade 1 LFT ^7^ ↑
Male	72	HCV	TARE:PR SBRT:SD	8	7/8	No	A	A	1	2	284.6	671.5	45	Grade 1 INR ^8^ ↑
Male	82	NAFLD, alcohol	TARE:SD SBRT:PR	2/4	7	No	A	B	2	1	316	653.5	45	Grade 1 ALP ↑
Female	40	NAFLD	TARE: PD SBRT: CR	3 and 6	5	No	A	A	0	2	211.2	888	45	None
Male	87	NAFLD, alcohol	TARE:PR SBRT:CR	8	8	Yes	A	A	1	2	439	1892	45	None

^1^ Creatinine, ^2^ Alkaline phosphatase, ^3^ Aspartate aminotransferase, ^4^ Bilirubin, ^5^ Albumin, ^6^ Alanine aminotransferase, ^7^ Liver function test, ^8^ International normalized ratio, ↑ Indicates post-treatment increase compared to baseline values.

## Data Availability

The data presented in this study are available on request from the corresponding author.

## Abbreviations

The following abbreviations are used in this manuscript:

HCC	Hepatocellular carcinoma
TARE	Transarterial radioembolization
SBRT	Stereotactic body radiation therapy
CP	Child-Pugh
TACE	Transarterial chemoembolization
ECOG	Eastern Cooperative Oncology Group
BCLC	Barcelona Clinic Liver Cancer
